# Peripheral blood metabolic and inflammatory factors as biomarkers to ocular findings in diabetic macular edema

**DOI:** 10.1371/journal.pone.0173865

**Published:** 2017-03-22

**Authors:** Marc Figueras-Roca, Blanca Molins, Anna Sala-Puigdollers, Jessica Matas, Irene Vinagre, José Ríos, Alfredo Adán

**Affiliations:** 1 Institut Clínic d'Oftalmologia (ICOF), Hospital Clínic, Barcelona, Spain; 2 August Pi i Sunyer Biomedical Research Institute (IDIBAPS), Barcelona, Spain; 3 Department of Endocrinology, Hospital Clínic, Barcelona, Spain; 4 Medical Statistics Core Facility, IDIBAPS, Barcelona, Spain; 5 Biostatistics Unit, Faculty of Medicine, Universitat Autònoma de Barcelona, Barcelona, Spain; Massachusetts Eye & Ear Infirmary, Harvard Medical School, UNITED STATES

## Abstract

**Aims:**

To study the association between peripheral blood metabolic and inflammatory factors and presence of diabetic macular edema (DME) and its related anatomic features in type 2 diabetic mellitus (T2DM) patients.

**Material and methods:**

Observational cross-sectional study on a proof of concept basis. Seventy-six T2DM included patients were divided based on the presence (n = 58) or absence of DME (n = 18) according to optical coherence tomography (OCT). Ultra-widefield fluorescein angiography (UWFA) was performed in DME patients. Fasting peripheral blood sample testing included glycemia, glycated hemoglobin, creatinin and lipid levels among others. Serum levels of a broad panel of cytokines and inflammatory mediators were also analysed. OCT findings included central subfoveal thickness, diffuse retinal thickness (DRT), cystoid macular edema (CME), serous retinal detachment and epirretinal membrane. UWFA items included pattern of DME, presence of peripheral retinal ischemia and enlarged foveal avascular zone (FAZ).

**Results:**

Metabolic and inflammatory factors did not statistically differ between groups. However, several inflammatory mediators did associate to certain ocular items of DME cases: IL-6 was significantly higher in patients with DRT (p = 0.044), IL-10 was decreased in patients with CME (p = 0.012), and higher IL-8 (p = 0.031) and VEGF levels (p = 0.031) were observed in patients with enlarged FAZ.

**Conclusion:**

Inflammatory and metabolic peripheral blood factors in T2DM may not be differentially associated to DME when compared to non-DME cases. However, some OCT and UWFA features of DME such as DRT, CME and enlarged FAZ may be associated to certain systemic inflammatory mediators.

## Introduction

Diabetic retinopathy (DR) is the most common microvascular complication of Diabetes Mellitus (DM) and is characterized by progressive retinal microvascular changes leading to tissue ischemia, increased permeability, neovascularization and edema.[1] If such changes affect the central area of the retina (macula), diabetic macular edema (DME) is developed. DME is the leading cause of decreased visual acuity in patients with DR, and its overall prevalence in DM patients is about 6.8%~14%.[2–4] Natural history of DME can cause significant vision loss in up to 50% of affected patients at two years time.[5] Type 2 DM (T2DM) is the most prevalent form of DM and about 3% of them do present DME 5 years after being diagnosed, but such a proportion dramatically increases with disease duration, reaching 28% at 20 years time.[6]

The pathogenesis of DME in T2DM is thought to be associated with increased vascular permeability due to breakdown of the blood–retinal barrier (BRB) and the blood–aqueous barrier. This breakdown is driven by the inflammation and oxidative stress produced by high levels of advanced glycation end-products.[7] Such events present with characteristic cellular and functional changes due to inflammation taking place in DR: leukostasis, abnormal leuckocyte adherence and increased permeability of retinal vascular barriers.[8] This global systemic inflammatory environment has been widely studied in diabetic patients. [8–12] However, it remains unclear whether the pathophysiology of DME is mainly attributed to such systemic affection or to a local (intraocular) response.

Several studies have published reports on systemic levels of metabolic and inflammatory mediators in DR but little is specifically known on DME. DR has been associated to higher serum levels of several cytokines, chemokines, growth and angiogenic factors such as interleukin (IL)-1β, IL-6, IL-8, monocyte chemoattractant protein-1 (MCP-1), tumor necrosis factor-alpha (TNF-α) and vascular endothelial growth factor (VEGF).[13–15] Metabolic and cardiovascular-related factors, such as poor glycemic control, blood hypertension, microalbuminuria, pregnancy, dyslipidemia, smoking habit and sedentary living have been also associated to DR development and progression.[11] However, there are only few studies focusing on systemic metabolic factors in DME, [16–19] and systemic inflammatory mediators in DME have not been studied so far. Inflammatory and vascular mediators in DME have been only studied locally in its intraocular setting. Indeed, it has been shown that DME patients have increased levels of pro-inflammatory mediators in aqueous humour (AH) compared to non-DME cases. [20] It is therefore controversial whether DME is also specifically associated to certain serum inflammatory and metabolic factors.

Finally, current diagnostic techniques on DME have greatly evolved from those used in past studies on systemic factors related to this entity. Optical coherence tomography (OCT) is a light-based imaging technique with no side events that allows tomographic reconstruction of any tissue, enabling, for example, characterization of cysts and extracellular edema within the macula. Ultra-widefield fluorescein angiography (UWFA) enhances visualization of retinal vessels and tissue ischemia up to 200° of photography of the retina (compared to the standard form of 45°). Both techniques have not been widely used in previous studies on this topic.

Given the current state of understanding of the complex systemic inflammatory and metabolic events in DM, we focused our research on DME. Although much reported information exists on differentiated levels of intraocular mediators of inflammation in diabetic patients with DME, little is known regarding such molecules in peripheral blood. Moreover, some reports on DME and its association to systemic items have not used current modern imaging techniques to identify DME such as OCT and UWFA. Therefore, the goal of the present study was to describe the association between peripheral blood metabolic and inflammatory factors and presence of DME and its related anatomic features in T2DM patients.

## Material and methods

### Study design

We present an observational cross-sectional study assessing serum levels of inflammatory and metabolic mediators in T2DM patients with and without DME.

The Hospital Clinic of Barcelona Institutional Review Board (IRB) approved this study according to local and national IRB guidelines. All DME and non-DME patients provided written informed consent, and the research followed the tenets of the Declaration of Helsinki. Eighty consecutive patients were screened when first attended in the Eye Department (Hospital Clínic of Barcelona, Spain) after primary care ophthalmologist referral because of DR with presumptive DME. Exclusion criteria was limited to proliferative DR (PDR), unclear ocular media, other retinal vasculopathy (such as retinal arterial or vein occlusion), pregnacy, systemic diseases of autoimmunitary or infectious condition, and systemic immunosupressive treatment of any kind. Whenever bilateral DME was present, the eye with the thickest edema in the fovea according to OCT measurements in microns was selected as the study eye.

All assessments and explorations, as well as peripheral blood extraction, were carried out the same day and in the same localiton; only UWFA could be delayed up to one week since inclusion date. Clinical assessment involved a complete medical and treatment history including DM duration.

### Cytokine and metabolic profile determination

Peripheral blood extraction was carried out at fasting time and included two samples simultaneously collected to determine serum levels of metabolic parameters and inflammatory markers respectively. For serum obtention, blood was centrifugated at 1600g within 30 minutes after obtention and stored at -70°C until inflammatory mediators were determined.

Searched metabolic parameters included glycemia, creatinin, total cholesterol, low-density lipoproteins cholesterol (LDL-C), high-density lipoproteins cholesterol (HDL-C), triglycerides (TG), aspartat aminotransferase (AST), alanine aminotransferase (ALT), hemoglobin (Hb) and glycated Hb (HbA_1C_). Such parameters analyses were carried out in regular facilities of the study-based terciary referral center. Ten immune mediators were determined: proinflammatory molecules such as IL-1β, IL-3, IL-6, IL-8, MCP-1, interferon gamma-induced protein-10 (IP-10); type 1 cytokines such as interferon gamma (IFN-γ) and TNF-α; type 2 cytokines (IL-10); and growth factors (VEGF). These molecules were chosen based on published results of previous studies regarding both serum and intraocular inflammatory biomarkers in DR and DME.[13–15, 20, 21] Selected immune mediators were determined by a Luminex platform (Millipore’s MilliPlex Human Cytokine/ Chemokine kit) used to measure cytokine and chemokine levels in serum samples using an assay plate layout consisting of seven standards in duplicate (3.2–2000 pg/mL), one blank well (for background fluorescence subtraction), two internal quality control samples in duplicate and 25 μL duplicates of each serum sample. The MilliPlex method was performed as recommended by the manufacturer.

### Ophthalmologic assessment

**Fig 1:**
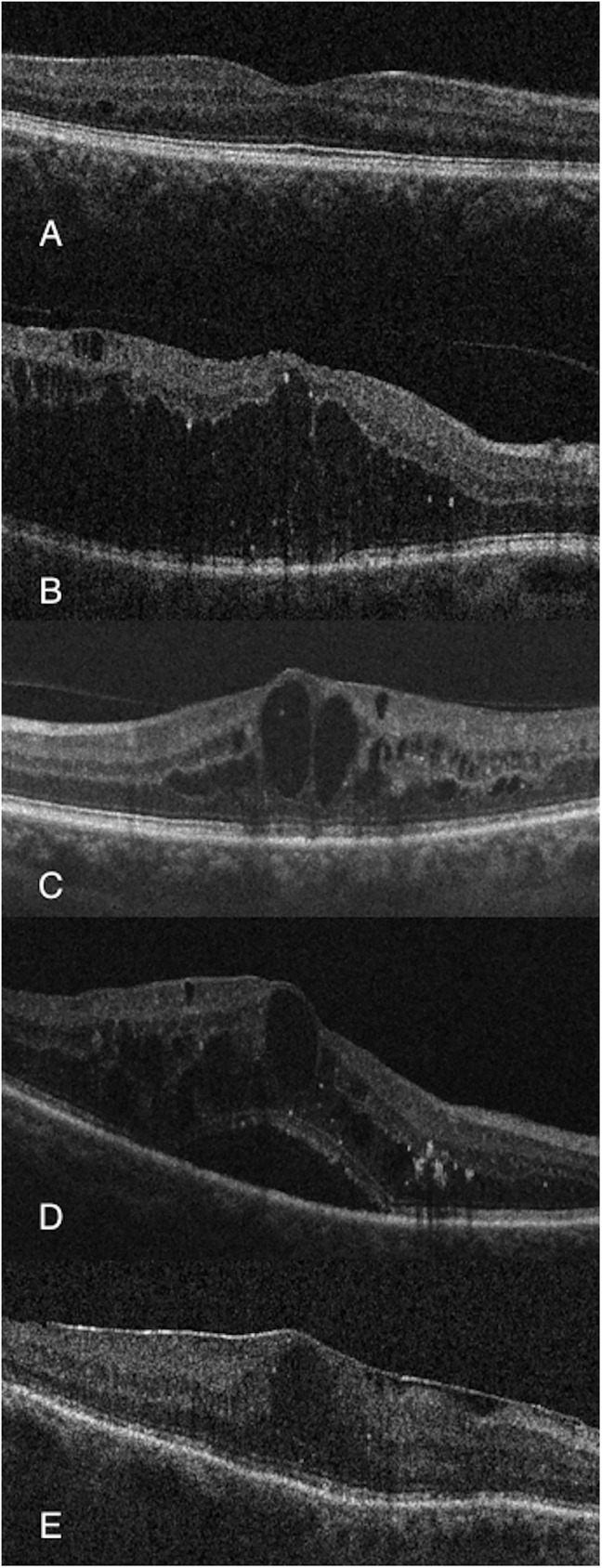
Examples of OCT-associated items. (A) No DME. (B) Diffuse retinal thickening. (C) Cystoid macular edema. (D) Serous retinal detachment. (E) Epirretinal membrane.

**Fig 2:**
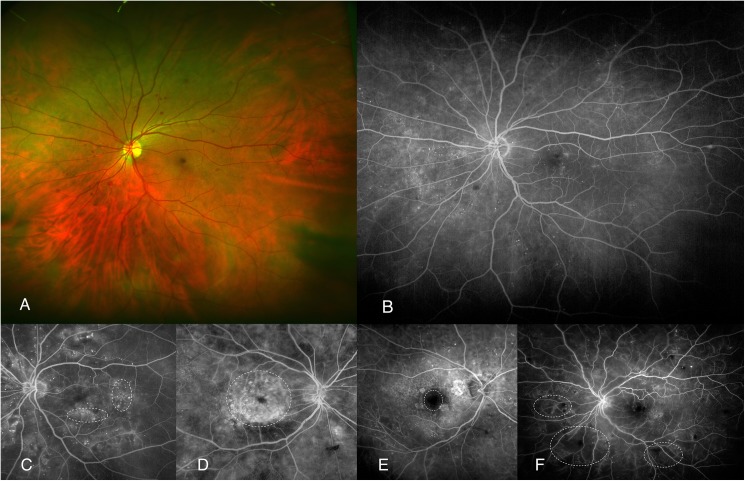
Examples of UWFA-based items (doted lines). (A) Color fundus photography of NPDR case without DME nor any associated sign. (B) UWFA of previous case. (C) focal-pattern DME. (D) diffuse-patern DME. (E) increased FAZ. (F) PRI.

Ophthalmologic assessment was also performed including past and current ophthalmic events and treatments and a complete eye examination was carried out with visual acuity determination, slit lamp biomicroscopy, intraocular pressure assessment and funduscopy under pupil dilation. Eye fundus exploration included DR grading according to international-based scale:[1, 22] mild non-proliferative DR (NPDR), moderate NPDR, severe NPDR and PDR. All subjects underwent macular OCT exploration (Cirrus, Carl-Zeeis Meditec, Inc, Dublin, CA) as gold-standard determination of DME using a standard Macular Cube 518x128 μm scan. Those diagnosed of DME were in addition studied with UWFA (Optomap, Optos PLC, Dunfermline, Scotland, UK). OCT findings included quantitative determination of central subfoveal thickness (CST) and qualitative findings (Fig 1) as presence of diffuse retinal thickening (DRT), cystoid macular edema (CME), serous retinal detachment (SRD) and epirretinal membrane (ERM). DME was OCT-confirmed when one of the next items was found:[23] CST>250μm, DRT, CME and/or SRD. UWFA findings (Fig 2) included: diffuse or focal pattern of DME according to common practice criteria [24] and previously published reports;[25] increased foveal avascular zone (FAZ) as defined by prior studies (>0.32mm^2^);[25] and presence of peripheral retinal ischemia (PRI) set as areas of capillary nonperfusion outside retinal vascular arcades.

### Statistical analysis

Description of variables were showed by mean, median, interquartilic range (percentiles 25^th^ and 75^th^) and absolute range for quantitative variables. Absolute frequencies and percentages were used in the description of qualitative factors. Inferential analyses were performed using Mann-Whitney U test. A billateral type I error of 5% was stablished. Analyses were first performed comparing DME patients with non-DME patients. On the other hand, analyses were carried out within the DME group of patients based on qualitative endpoints (for example, comparing CME cases with non-CME ones). Due to observational characteristics of the present study a strategy for multiplicity adjust was not planned, thus significant results should be validated in posterior independent studies. All statistical calculations were executed on SPSS v.20.0 (SPSS IBM Corporation, New York, NY, USA).

## Results

**Fig 3:**
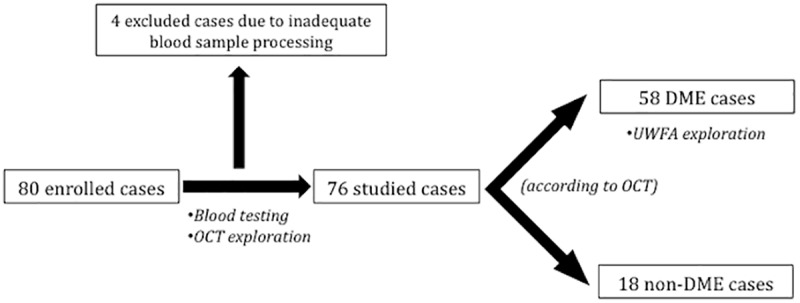
Flowchart of the study design.

Eighty DM patients were included in the study according to previously stated criteria. Four patients were finally excluded due to blood sample processing malfunction and no possible determination of inflammatory markers. Seventy-six patients were finally analysed. Fifty-eight of them presented DME according to OCT-based defined endpoints. Eighteen patients referred for suspected DME did not achieve such endpoint and therefore constituted the non-DME group. All included patients had peripheral venous blood extraction and OCT exploration made. Of 58 DME patients, 42 subjects (72%) underwent UWFA according to study protocol; 16 patients did not undergo UWFA due to delayed planning or refusal (Fig 3). Demographics of both groups (Table 1) reported no differences between age, gender and DM duration. Regarding DR grading, all non-DME cases (n = 18) showed signs of mild NPDR whereas DME cases presented mild NPDR in 25 cases (43%), moderate NPDR in 22 subjects (38%) and severe NPDR in 11 cases (19%).

**Table 1:** Clinical parameters of patients with and without DME.

	No DME (n = 18)	DME (n = 58)	p-value
**Demographics:**			
Age (years)	65 [59; 76]	70 [62; 78]	0.264
	32 to 88	53 to 85	
Gender			
Male (%)	13 (72.2%)	34 (58.6%)	0.408
Female (%)	5 (27.8%)	24 (41.4%)	
DM duration (years)	11 [8; 14]	11 [8; 19]	0.344
	1 to 25	4 to 40	
**Serum metabolic markers:**			
Glucose (mg/dl)	191 [128; 264]	146 [116; 213]	0.220
	62 to 341	51 to 417	
Creatinine (mg/dl)	0.84 [0.68; 0.96]	0.87 [0.77; 1.03]	0.330
	0.54 to 1.34	0.37 to 4.13	
Total cholesterol (mg/dl)	184 [148; 200]	172 [150; 193]	0.545
	95 to 281	90 to 249	
Triglyceride (mg/dl)	116 [85; 204]	112 [85; 172]	0.648
	49 to 832	43 to 359	
AST (U/L)	20 [17; 28]	20 [16; 26]	0.402
	15 to 69	8 to 78	
ALT (U/L)	22 [19; 28]	21 [15; 29]	0.250
	15 to 106	6 to 119	
LDL-C (mg/dl)	106 [79; 128]	99 [76; 119]	0.641
	56 to 159	38 to 175	
HDL-C (mg/dl)	40 [34; 47]	44 [37; 59]	0.104
	22 to 68	24 to 84	
Hb (mg/dl)	145 [127; 155]	136 [124; 148]	0.288
	119 to 159	98 to 175	
HbA1C (%)	8.3 [8; 9.4]	7.7 [6.9; 8.9]	0.082
	6.1 to 13.3	5.5 to 13.8	
**Serum inflammatory markers:**			
IL-1β (pg/ml)	0.8 [0.8; 0.8]	0.8 [0.8; 0.8]	0.753
	0.8 to 3.71	0.8 to 6.90	
IL-3 (pg/ml)	0.7 [0.7; 0.7]	0.7 [0.7; 0.7]	1
	0.7 to 0.7	0.7 to 162.15	
IL-6 (pg/ml)	0.9 [0.9; 1.53]	0.9 [0.9; 0.9]	0.258
	0.9 to 25.91	0.9 to 81.14	
IL-8 (pg/ml)	8.23 [5.38; 10.72]	8.78 [4.19; 12.60]	0.842
	2.06 to 25.19	0.82 to 108.03	
IL-10 (pg/ml)	1.1 [1.1; 1.1]	1.1 [1.1; 1.1]	0.741
	1.1 to 33.74	1.1 to 12.12	
MCP-1 (pg/ml)	401.73 [323.08; 458.65]	421.29 [361.56; 547.26]	0.465
	249.65 to 656.79	143.94 to 1115.98	
IP-10 (pg/ml)	254.95 [189.66; 378.41]	206.6 [140.91; 366.42]	0.266
	128.54 to 808.90	45.61 to 1271.69	
IFN-γ (pg/ml)	1.18 [0.8; 4.3]	0.89 [0.8; 3.42]	0.477
	0.8 to 58.31	0.8 to 572.05	
TNF-α (pg/ml)	5.74 [4.27; 8.19]	7.58 [5.23; 10.24]	0.078
	1.75 to 10.61	0.7 to 33.06	
VEGF (pg/ml)	26.3 [26.3; 293.59]	26.3 [26.3; 274.69]	0.631
	26.3 to 717.13	26.3 to 3269.42	

*Variables are described by median and interquartilic range [Percentiles 25*^*th*^, *75*^*th*^*] and absolute range*, *except from gender*.

*Abbreviations*: *ALT*, *alanine aminotransferase; AST*, *aspartat aminotransferase; Hb*, *hemoglobin; HbA1C*, *glycosylated hemoglobin-A1; HDL-C*, *high-density lipoproteins cholesterol; IL*, *interleukin; IP-10*, *interferon gamma-induced protein-10; IQR*, *interquartilic range; LDL-C*, *low-density lipoproteins cholesterol; MCP-1*, *monocyte chemoattractant protein-1; SD*, *standard deviation; TNF-*α, *tumour necrosis factor alpha; VEGF*, *vascular endothelial growth factor*.

No statistically significant differences were found between DME and non-DME patients on metabolic blood parameters (glycemia, creatinin, total cholesterol, LDL-C, HDL-C, TG, AST, ALT, Hb and HbA_1C_) and serum inflammatory biomarkers (IL-1β, IL-3, IL-6, IL-8, IL-10, MCP-1, IP-10, IFN-γ, TNF-α and VEGF) (Table 1).

Anatomic findings associated to DME as OCT and UWFA findings are shown in Table 2. A subanalysis of the DME patients was done according to an arbitrary split point of CST as an explorative clinical marker of mild (<450μm) or severe (≥450μm) DME presentation. Such split point was proposed by the authors on a proof of concept basis as an intended marker of DME severity. No statistically significant differences were found regarding blood parameters and inflammatory factors between such groups. However, BCVA (logMAR) did statistically significantly differ (p<0.001) between them with better visual acuity found in mild DME cases (median 0.3 [IQR: 0.1; 0.5]) thant severe ones (median 0.7 [IQR 0.5; 0.8]).

**Table 2:** OCT and UWFA findings of DME.

	n (%)
**OCT:**	
CST <450μm	33 (57)
CST ≥450μm	25(43)
Diffuse retinal thickening	22 (38)
Cystoid macular edema	38 (65)
Serous retinal detachment	7 (12)
Epirretinal membrane	4 (7)
**UWFA:**	
Diffuse-pattern DME	14 (33)
Focal-pattern DME	28 (66)
Increased foveal avascular zone	6 (14)
Peripheral retinal ischemia	11 (26)

*Abbreviations*: *CST*, *central subfoveal thickness; DME*, *diabetic macular edema; OCT*, *optical coherence tomography; UWFA*, *ultra-widefield fluorescein angiography*.

**Fig 4:**
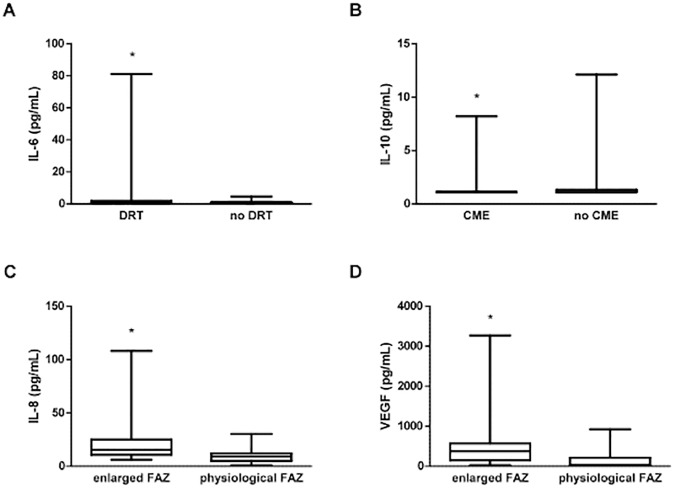
Boxplot graphic showing statistically significant differences (*) regarding peripheral blood inflammatory mediators and OCT and UWFA DME-associated items. (A) Increased IL-6 levels associated to DRT. (B) Decreased IL-10 levels related to CME. (C) Increased IL-8 and VEGF concentration (D) associated to enlarged FAZ.

Regarding further analysis within the DME group, metabolic and inflammatory markers were compared according to OCT and UWFA anatomic findings (S1 Table and S2 Table respectively). Statistically significant associations are reported in Table 3 and shown in Fig 4. DME patients presenting with DRT (n = 22) were therefore related (p = 0.044) to higher IL-6 peripheral blood serum levels. Cases of CME in the OCT exploration (n = 38) were associated (p = 0.012) to lower IL-10 concentration. Finally, significant differences were also found regarding DME patients with enlarged FAZ (n = 6) showing higher serum levels of IL-8 (p = 0.031) and VEGF (p = 0.031) when compared to those with physiological FAZ.

**Table 3:** Inflammatory factors associated to OCT and UWFA findings of DME.

	OCT and UWFA finding		p-value
	**DRT (n = 22)**	**no DRT (n = 36)**	
**IL-6** (ρg/ml)	6.57 (17.60)	1.09 (0.69)	0.044
	0.9 [0.9; 1.76]	0.9 [0.9; 0.9]	
	0.9 to 81.14	0.9 to 4.53	
	**CME (n = 38)**	**no CME (n = 20)**	
**IL-10** (ρg/ml)	1.29 (1.17)	2.28 (3.33)	0.012
	1.1 [1.1; 1.1]	1.1 [1.1; 1.32]	
	1.1 to 8.21	1.1 to 12.12	
	**enlarged FAZ (n = 6)**	**physiological FAZ (n = 36)**	
**IL-8** (ρg/ml)	30.17 (38.69)	9.64 (6.18)	0.031
	15.49 [10.73; 25.10]	9.05 [5.02; 12.01]	
	6.17 to 108.03	0.82 to 30.33	
**VEGF** (ρg/ml)	794.54 (1227.54)	158.55 (239.81)	0.031
	374.07 [152.32; 571.07]	26.3 [26.3; 211.52]	
	26.3 to 3269.42	26.3 to 925.66	

*Variables are described (above to lower) by mean and standard deviation*, *median and interquartilic range [percentiles 25*^*th*^, *75*^*th*^*]*, *and absolute range*.

*Abbreviations*: *CME*, *cystoid macular edema; DRT*, *diffuse retinal thickening; DME*, *diabetic macular edema; FAZ*, *foveal avascular zone; n*, *number of cases; OCT*, *optical coherence tomography; UWFA*, *ultra-widefield fluorescein angiography*.

## Discussion

This cross-sectional study aimed to analyse serum inflammatory and metabolic factors (cytokines, growth factors, and common practice metabolic items) and their association to DME in first place and, in addition, their relation to ophthalmic anatomic features studied by latest cutting-edge technology.

As a whole, no statistically significant differences were found in all parameters between the DME and the non-DME group (Table 1). At first glance, one could at least expect HbA_1C_ to be higher in the DME group according to the general understandig of this DM complication.[11, 17] However, studied non-DME patients were indeed referred patients with DR and no DME confirmed by fundus and OCT examination. Non-DME patients could therefore deploy a worse metabolic peripheral blood profile than similar non-DME DM outclinic patients not attending the primary care ophthalmologist. Therefore their comparision with DME patients could be inaccurate. Regarding serum inflammatory markers, one could expect some relationship with DME as intraocular levels are indeed increased in DME. [20] However, none of them did statistically differ between groups. Although levels of TNF-α showed a higher concentration in DME patients (median 7.58 ρg/ml) than non-DME ones (median 5.74 ρg/ml), such a difference did not match the established significance level (p = 0.078). A recent published study [26] reached similar conclusions when reporting increased serum levels of TNF-α in DME patients. However, such a finding was only statistically significant when comparing levels on DME patients to healthy non-DM controls. [26]. Moreover, this difference was not significant when comparing DME cases with either DR patients with no-DME, or DM patients with no DR. [26].

In the second part of the study we focused on the DME group to identify associations between certain clinical and anatomical characteristics of DME cases and systemic levels of several factors. We therefore selected defined endpoints that may influence DME prognosis and can be easily determined by objective explorations. BCVA, although a major clinical outcome of DME, was not one of these items since it can be easily biased by other parameters such as DME duration, presence of cataract and macular ischemia. However, in the present study, BCVA remained a good marker of internal validity as was significantly (p = 0.001) higher in mild DME cases (CST<450μm) than severe ones (CST≥450μm).

Regarding OCT-based items, we could first expect increased CST to be associated to higher levels of inflammatory serum mediators. Despite that, no differences were found regarding serum cytokines and other molecules between mild DME cases (CST<450μm) and severe ones (CST≥450μm). Such a finding may reinforce the idea that the systemic inflammatory milieu does not completely explain DME behaviour. The fact that DME patients present with mild or severe forms could be therefore related in fact to the intraocular concentration of such molecules. Some of the other OCT-based qualitative findings (DRT, CME, SRD and ERM) have been indeed interestingly associated to intraocular levels of inflammatory mediators. [27, 28] DME with DRT is thought to be related to poor glycemic control and poor response to treatment. Our analysis showed increased serum levels of IL-6 in patients with DRT (Table 3, Fig 4). IL-6 is a multifunctional cytokine essential for acute phase reactions and regulation of immune processes known to be involved in both increased vascular permeability and angiogenesis.[28–30] Such a finding could support the idea of a strong inflammatory nature of this macular feature. Moreover, high blood levels of IL-6 have already been reported to be associated to severe grades of DR itself,[14] commonly found together with DRT cases of DME in DM patients with poor glycemic control. Another studied item was CME, which stands for certain cases of DME presenting with major intraretinal cysts within inner retinal layers. Regarding CME, no differences between cytokines were found, except for IL-10, which was significantly decreased in the DME group (Table 3, Fig 4). IL-10 is a pleiotropic immunosuppressive and immunostimulatory cytokine which is able to down-regulate inflammatory activity of T lymphocytes helper 1.[20, 27, 31, 32] In addition, it also prevents angiogenesis by downregulating VEGF. [27, 32] To our knowledge, there have not been previous reports on systemic IL-10 in DR nor DME. However, some studies did report low AH concentration of IL-10 in DR and even DME [20, 32]. Our results would suggest that low IL-10 blood levels could be related to CME findings in DME. Finally, as OCT-based items, SRD and ERM were also studied. SRD stands for an anatomical subtype of edema with subretinal location of fluid caused by breakdown of the outer BRB. [27, 28] ERM, in turn, stands for epithelial proliferation on the inner surface of the retina. No differences were found between patients with and without these features, most likely due to the small number of cases with either SRD (n = 7) or ERM (n = 4).

Regarding UWFA findings, studied items included pattern of DME, enlarged FAZ and PRI. No significant differences were found between focal and diffuse DME pattern in any of the analysed markers. This could therefore enforce current theories on edema location within the macula being mainly driven by local factors instead of systemic ones. We also analysed two other items by UWFA: PRI and enlarged FAZ. PRI depicted by UWFA could be a way to characterize retinal microangiopathy and tissue ischemia in DM. PRI is nowadays a topic of major importance due to its presumed involvement in DME formation and maintenance; W*essel et al*. [33] found 83% of studied DME cases to be associated to PRI with a statistically significant odds ratio of 3.75 when compared to PRI in DR non-DME subjects. In our studied DME group, only 26% of DME cases did present with PRI on UWFA. More reports are needed to confirm this point. Regarding PRI, peripheral blood mediators did not match significance level.

The avascular zone of the central retina (fovea) is known as FAZ. An enlarged FAZ in DME patients is presumed to be associated to certain grades of macular ischemia and therefore poor visual prognosis. Moreover, FAZ itself has been directly correlated to PRI in some UWFA-based reports.[25] Interestingly, we found that peripheral blood levels of IL-8 and VEGF were statistically higher in enlarged FAZ DME cases (Table 3, Fig 4). The pro-inflammatory chemokine IL-8 is a major attractant and activator of neutrophils and T lymphocites. Its increased levels have been associated to advanced stages of DR such as PDR due to vessel gliotic obliteration.[32]. VEGF, perhaps the most studied and well-known growth factor in DR, is an endothelial cell mitogen inducing vascular permeability and angiogenesis.[32] Increased IL-8 and VEGF in DME cases with enlarged FAZ could suggest central retinal vasculopathy cases to be somehow systemic-related. However, lack of statistical significance regarding PRI findings does not allow a better knowledge on how serum inflammatory mediators associate to DME and local vascular-related events.

As a final point of discussion it is mandatory to take into consideration the limitations of the present investigation. Although designed on a proof of concept basis, the cross-sectional nature of the study limits the extent of the conclusions. Reduced sample size and variability of cytokines and growth factors remain as main limitations. In addition, lack of correction for multiple comparisons could affect the study power to detect differences. Therefore, our findings should be validated in posterior independent studies and the reported associations should be carefully considered. More studies are warranted with increased group sizes in order to further understand the involvement of systemic mediators in DME.

### Conclusion

The results of our study suggest that inflammatory and metabolic serum mediators are not differentially associated to DME when compared to non-DME patients. However, we do report several OCT and UWFA features of DME such as DRT, CME and enlarged FAZ to be associated to certain systemic inflammatory mediators. Given that DME is a multifactorial condition resulting from complex interactions of both systemic and local inflammatory events, such findings could be of major importance as a starting point to further investigate the contribution of systemic inflammation in DME.

## Supporting information

S1 Table Inflammatory mediators associated to OCT findings of DME. (DOC)

S2 Table Inflammatory mediators associated to UWFA findings of DME. (DOC)
