# Supplementary material for: Peripheral blood metabolic and inflammatory factors as biomarkers to ocular findings in diabetic macular edema
Source: PLoS One. 2017 Mar 22;12(3):e0173865. doi: 10.1371/journal.pone.0173865 (PMC5362077; doi:10.1371/journal.pone.0173865)
Supplement: S1 Table — (DOC) [file pone.0173865.s001.doc]

S1 Table. Inflammatory mediators associated to OCT findings of DME

| **OCT and UWFA finding** | **IFN-γ** (ρg/ml) | **IL-1β** (ρg/ml) | **IL-3** (ρg/ml) | **IL-6** (ρg/ml) | **IL-8** (ρg/ml) | **IL-10** (ρg/ml) | **MCP-1** (ρg/ml) | **IP-10** (ρg/ml) | **TNF-α** (ρg/ml) | **VEGF** (ρg/ml) |
| --- | --- | --- | --- | --- | --- | --- | --- | --- | --- | --- |
| **CME** (n=38) | 19.13 (93.86) | 1.02 (1.06) | 5.06 (26.54) | 4.27 (13.59) | 10.89 (17.28) | 1.29 (1.17) | 450.88 (180.29) | 272.43 (169.20) | 7.67 (4.00) | 245.33 (561.05) |
|  | 0.89 [0.8; 2.62] | 0.8 [0.8; 0.8] | 0.7 [0.7; 0.7] | 0.9 [0.9; 0.9] | 6.82 [4.19; 10.987] | 1.1 [1.1; 1.1] | 421.30 [366.92; 543.99] | 212.14 [147.56; 366.42] | 7.19 [5.0246; 9.21] | 26.3 [26.3; 213.33] |
|  | 0.8 to 572.05 | 0.8 to 6.90 | 0.7 to 162.15 | 0.9 to 81.15 | 1.51 to 108.04 | 1.1 to 8.21 | 143.95 to 1115.98 | 71.24 to 750.82 | 0.98 to 18.82 | 26.3 to 3269.42 |
| **no CME** (n=20) | 5.6284 (8.50) | 1.22 (1.34) | 0.7 (0) | 1.15 (0.84) | 11.13 (7.57) | 2.29 (3.34) | 474.10 (168.95) | 294.63 (285.57) | 9.45 (6.98) | 253.58 (257.65) |
|  | 1.85 [0.8; 6.27] | 0.8 [0.8; 0.8] | 0.7 [0.7; 0.7] | 0.9 [0.9; 0.9] | 9.31 [7.21; 14.911] | 1.1 [1.1; 1.32] | 448.62 [361.35; 579.10] | 199.6 [115.4; 377.60] | 8.38 [6.10; 10.53] | 168.53 [26.3; 391.42] |
|  | 0.8 to 32.28 | 0.8 to 6.19 | 0.7 to 0.7 | 0.9 to 4.61 | 0.82 to 30.34 | 1.1 to 12.13 | 224.69 to 887.96 | 45.62 to 1271.70 | 0.7 to 33.07 | 26.3 to 736.17 |
| **p-value** | 0.416 | 0.5 | 1 | 0.645 | 0.274 | 0.012 | 0.621 | 0.548 | 0.313 | 0.178 |
| **DRT** (n=22) | 32.78 (124.20) | 1.34 (1.51) | 8.39 (35.23) | 6.57 (17.60) | 13.26 (22.63) | 2.02 (2.75) | 434.54 (167.82) | 257.37 (193.88) | 9.66 (6.26) | 374.75 (712.78) |
|  | 0.89 [0.8; 3.65] | 0.8 [0.8; 0.8] | 0.7 [0.7; 0.7] | 0.9 [0.9; 1.76] | 8.26 [4.39; 10.99] | 1.1 [1.1; 1.1] | 394.10 [361.57; 544.48] | 201.11 [136.88; 362.31] | 7.76 [7.03; 10.24] | 184.73 [26.3; 368.86] |
|  | 0.8 to 572.05 | 0.8 to 6.90 | 0.7 to 162.15 | 0.9 to 81.14 | 1.51 to 108.04 | 1.1 to 11.91 | 143.95 to 854.73 | 45.62 to 750.82 | 3.71 to 33.07 | 26.3 to 3269.42 |
| **no DRT** (n=36) | 3.68 (5.85) | 0.95 (0.90) | 0.7 (0) | 1.09 (0.69) | 9.64 (6.37) | 1.42(1.84) | 472.41 (179.65) | 293.55 (227.87) | 7.50 (4.47) | 174.43 (233.05) |
|  | 1.00 [0.8; 3.02] | 0.8 [0.8; 0.8] | 0.7 [0.7; 0.7] | 0.9 [0.9; 0.9] | 9.29 [4.02; 13.65] | 1.1 [1.1; 1.1] | 468.31 [365.57; 556.77] | 223.19 [148.65; 371.63] | 7.44 [4.72; 9.73] | 26.3 [26.3; 235.68] |
|  | 0.8 to 24.55 | 0.8 to 6.19 | 0.7 to 0.7 | 0.9 to 4.53 | 0.82 to 25.4 | 1.1 to 12.13 | 224.69 to 1115.98 | 77.17 to 1271.69 | 0.7 to 19.69 | 26.3 to 754.54 |
| **p-value** | 0.667 | 0.106 | 0.368 | 0.044 | 0.655 | 0.138 | 0.445 | 0.407 | 0.247 | 0.201 |
| **SRD** (n=7) | 9.01 (19) | 1.67 (2.31) | 0.7 (0) | 2.06 (2.71) | 6.27 (4.94) | 1.1 (0) | 413.94 (76.06) | 262.07 (240.35) | 7.64 (2.25) | 297.14 (374.25) |
|  | 2.28 [0.8; 3.76] | 0.8 [0.8; 0.8] | 0.7 [0.7; 0.7] | 0.9 [0.9; 1.77] | 4.39 [2.77; 10.99] | 1.1 [1.1; 1.1] | 415.47 [374.18; 421.30] | 189.98 [89.06; 392.89] | 7.12 [5.89; 8.92] | 26.3 [26.3; 680.29] |
|  | 0.8 to 52.02 | 0.8 to 6.90 | 0.7 to 0.7 | 0.9 to 8.16 | 1.5141 to 15.16 | 1.1 to 1.1 | 312.76 to 544.48 | 71.241 to 750.82 | 5.30 to 12.03 | 26.3 to 925.66 |
| **no SRD** (n=51) | 15.15 (80.63) | 1.01 (0.92) | 3.93 (22.83) | 3.31 (11.68) | 11.64 (15.33) | 1.72 (2.35) | 464.88 (183.24) | 282.76 (213.68) | 8.39 (5.55) | 241.38 (489.37) |
|  | 0.89 [0.8; 3.42] | 0.8 [0.8; 0.8] | 0.7 [0.7; 0.7] | 0.9 [0.9; 0.9] | 9.00 [4.69; 12.91] | 1.1 [1.1; 1.1] | 439.39 [361.57; 558.83] | 215.49 [146.38; 366.42] | 7.73 [5.02; 10.24] | 26.3 [26.3; 243.22] |
|  | 0.8 to 572.05 | 0.8 to 6.19 | 0.7 to 162.15 | 0.9 to 81.15 | 0.82 to 108.04 | 1.1 to 12.13 | 143.95 to 1115.98 | 45.62 to 1271.70 | 0.7 to 33.07 | 26.3 to 3269.42 |
| **p-value** | 0.664 | 0.156 | 1 | 0.618 | 0.174 | 0.591 | 0.645 | 0.481 | 0.807 | 0.771 |
| **ERM** (n=4) | 4.29 (3.57) | 0.8 (0) | 0.7 (0) | 1.38 (0.96) | 11.61 (10.218) | 1.1 (0) | 517.19 (196.83) | 331.51 (129.49) | 7.75 (3.97) | 196.51 (210.41) |
|  | 3.62 [1.74; 6.84] | 0.8 [0.8; 0.8] | 0.7 [0.7; 0.7] | 0.9 [0.9; 1.86] | 9.66 [4.21; 19] | 1.1 [1.1; 1.1] | 578.45 [390.20; 644.17] | 339.11 [220.23; 442.80] | 9.00 [4.92; 10.57] | 150.50 [26.3; 366.71] |
|  | 0.8 to 9.12 | 0.8 to 0.8 | 0.7 to 0.7 | 0.9 to 2.8117 | 1.70 to 25.4 | 1.1 to 1.1 | 233.14 to 678.71 | 201.11 to 446.73 | 2.16 to 10.82 | 26.3 to 458.72 |
| **no ERM** (n=54) | 15.16 (78.50) | 1.12 (1.20) | 3.75 (22.177) | 3.29 (11.37) | 10.93 (14.89) | 1.68 (2.29) | 454.79 (174.57) | 276.35 (220.35) | 8.34 (5.36) | 252.13 (489.31) |
|  | 0.89 [0.8; 2.96] | 0.8 [0.8; 0.8] | 0.7 [0.7; 0.7] | 0.9 [0.9; 0.9] | 8.78 [4.19; 12.02] | 1.1 [1.1; 1.1] | 417.72 [361.57; 543.99] | 203.85 [138.44; 362.31] | 7.55 [5.23; 9.21] | 26.3 [26.3; 243.22] |
|  | 0.8 to 572.05 | 0.8 to 6.90 | 0.7 to 162.15 | 0.9 to 81.15 | 0.82 to 108.04 | 1.1 to 12.1271 | 143.95 to 1115.98 | 45.62 to 1271.70 | 0.7 to 33.07 | 26.3 to 3269.42 |
| **p-value** | 0.262 | 1 | 1 | 0.82 | 0.796 | 1 | 0.245 | 0.227 | 0.78 | 0.854 |
| - *Variables are described (above to lower) by mean and standard deviation, median and interquartilic range [percentiles 25^th^, 75^th^], and absolute range* - *Abbreviations: CME, cystoid macular edema; DRT, diffuse retinal thickening; DME, diabetic macular edema; ERM, epirretinal membrane; OCT, optical coherence tomography.* | | | | | | | | | | |
