# Supplementary material for: Peripheral blood metabolic and inflammatory factors as biomarkers to ocular findings in diabetic macular edema
Source: PLoS One. 2017 Mar 22;12(3):e0173865. doi: 10.1371/journal.pone.0173865 (PMC5362077; doi:10.1371/journal.pone.0173865)
Supplement: S2 Table — (DOC) [file pone.0173865.s002.doc]

S2 Table. Inflammatory mediators associated to UWFA findings of DME

| **OCT and UWFA finding** | **IFN-γ** (ρg/ml) | **IL-1β** (ρg/ml) | **IL-3** (ρg/ml) | **IL-6** (ρg/ml) | **IL-8** (ρg/ml) | **IL-10** (ρg/ml) | **MCP-1** (ρg/ml) | **IP-10** (ρg/ml) | **TNF-α** (ρg/ml) | **VEGF** (ρg/ml) |
| --- | --- | --- | --- | --- | --- | --- | --- | --- | --- | --- |
| **Focal DME** (n=28) | 22.82 (107.74) | 1.08 (1.09) | 6.47 (30.51) | 4.48 (15.52) | 13.61 (19.89) | 1.76 (2.40) | 503.74 (201.06) | 311.99 (259.48) | 8.79 (6.60) | 280.29 (625.35) |
|  | 0.8 [0.8; 2.08] | 0.8 [0.8; 0.8] | 0.7 [0.7; 0.7] | 0.9 [0.9; 0.9] | 9.32 [4.54; 13.40] | 1.1 [1.1; 1.1] | 468.31 [384.80; 579.10] | 209.41 [149.24; 402.65] | 7.5677 [4.79; 10.73] | 43.18 [26.3; 220.73] |
|  | 0.8 to 572.05 | 0.8 to 6.19 | 0.7 to 162.15 | 0.9 to 81.15 | 0.82 to 108.04 | 1.1 to 11.91 | 224.69 to 1115.98 | 78.20 to 1271.70 | 0.98 to 33.065 | 26.3 to 3269.42 |
| **Diffuse DME** (n=14) | 7.46 (14.32) | 1.31 (1.76) | 0.7 (0) | 1.80 (2.09) | 10.66 (4.04) | 1.14 (0.13) | 409.15 (170.18) | 288.54 (189.88) | 8.92 (4.69) | 203.50 (286.23) |
|  | 3.22 [0.8; 6.39] | 0.8 [0.8; 0.8] | 0.7 [0.7; 0.7] | 0.9 [0.9; 1.72] | 10.02 [7.49; 13.77] | 1.1 [1.1; 1.1] | 411.41 [248.19; 543.99] | 232.19 [169.55; 404.47] | 8.81 [6.02; 11.42] | 26.3 [26.3; 304.43] |
|  | 0.8 to 52.02 | 0.8 to 6.90 | 0.7 to 0.7 | 0.9 to 8.16 | 5.03 to 18.07 | 1.1 to 1.54 | 143.95 to 678.71 | 77.170 to 750.82 | 0.7 to 18.82 | 26.3 to 925.66 |
| **p-value** | 0.097 | 0.694 | 1 | 0.347 | 0.512 | 0.677 | 0.286 | 0.942 | 0.497 | 0.857 |
| **Enlarged FAZ** (n=6) | 97.80 (232.3) | 1.19 (0.95) | 27.61 (65.91) | 14.40 (32.70) | 30.17 (38.70) | 2.38 (2.87) | 521.31 (236.17) | 340.76 (126.93) | 11.71 (6.69) | 794.55 (1227.55) |
|  | 2.60 [1.12; 7.64] | 0.8 [0.8; 0.8] | 0.7 [0.7; 0.7] | 0.9 [0.9; 1.68] | 15.49 [10.73; 25.10] | 1.1 [1.1; 1.68] | 540.41 [299.64; 626.93] | 322.07 [260.74; 437.93] | 12.86 [7.59; 17.39] | 374.07 [152.33; 571.08] |
|  | 0.8 to 572.05 | 0.8 to 3.12 | 0.7 to 162.15 | 0.9 to 81.15 | 6.17 to 108.04 | 1.1 to 8.22 | 232.51 to 887.96 | 176.86 to 524.91 | 0.7 to 18.82 | 26.3 to 3269.42 |
| **Physiological FAZ** (n=36) | 4.09 (9.44) | 1.13 (1.36) | 0.7 (0) | 1.68 (2.47) | 9.64 (6.19) | 1.42 (1.83) | 465.84 (188.31) | 294.76 (251.31) | 8.26 (5.79) | 158.55 (239.82) |
|  | 0.8 [0.8; 3.42] | 0.8 [0.8; 0.8] | 0.7 [0.7; 0.7] | 0.9 [0.9; 0.9] | 9.05 [5.03; 12.02] | 1.1 [1.1; 1.1] | 423.44 [370.01; 566.27] | 203.64 [146.38; 371.00] | 7.55 [4.82; 10.24] | 26.3 [26.3; 211.52] |
|  | 0.8 to 52.02 | 0.8 to 6.90 | 0.7 to 0.7 | 0.9 to 13.22 | 0.82 to 30.34 | 1.1 to 11.91 | 143.95 to 1115.98 | 77.17 to 1271.70 | 0.98 to 33.07 | 26.3 to 925.66 |
| **p-value** | 0.109 | 0.386 | 0.146 | 0.405 | 0.031 | 0.066 | 0.517 | 0.183 | 0.118 | 0.031 |
| **PRI** (n=11) | 58.68 (170.93) | 1.57 (1.90) | 15.38 (48.68) | 9.01 (24.02) | 20.49 (29.66) | 1.75 (2.15) | 501.82 (206.86) | 418.79 (349.08) | 8.68 (5.20) | 510.96 (952.87) |
|  | 2.23 [0.8; 7.64] | 0.8 [0.8; 0.8] | 0.7 [0.7; 0.7] | 0.9 [0.9; 1.77] | 10.99 [6.38; 18.07] | 1.1 [1.1; 1.1] | 488.11 [299.64; 661.33] | 281.82 [176.86; 527.06] | 9.21 [4.75; 12.73] | 152.33 [26.3; 413.98] |
|  | 0.8 to 572.05 | 0.8 to 6.90 | 0.7 to 162.15 | 0.9 to 81.15 | 3.84 to 108.04 | 1.1 to 8.22 | 232.51 to 887.96 | 79.94 to 1271.70 | 0.7 to 17.39 | 26.3 to 3269.42 |
| **no PRI** (n=31) | 2.8146 (4.75) | 0.98 (0.98) | 0.7 (0) | 1.53 (2.37) | 9.77 (6.50) | 1.49 (1.97) | 464.94 (192.04) | 258.49 (167.47) | 8.79 (6.31) | 156.53 (225.21) |
|  | 0.85 [0.8; 2.9638] | 0.8 [0.8; 0.8] | 0.7 [0.7; 0.7] | 0.9 [0.9; 0.9] | 9.16 [5.03; 12.02] | 1.1 [1.1; 1.1] | 419.40 [370.01; 547.26] | 205.27 [146.38; 362.31] | 7.64 [5.02; 10.24] | 26.3 [26.3; 211.52] |
|  | 0.8 to 24.55 | 0.8 to 6.19 | 0.7 to 0.7 | 0.9 to 13.22 | 0.82 to 30.34 | 1.1 to 11.91 | 143.95 to 1115.98 | 77.17 to 733.32 | 0.98 to 33.07 | 26.3 to 754.54 |
| **p-value** | 0.188 | 0.119 | 0.268 | 0.142 | 0.174 | 0.89 | 0.469 | 0.183 | 0.67 | 0.137 |
| - *Variables are described (above to lower) by mean and standard deviation, median and interquartilic range [percentiles 25^th^, 75^th^], and absolute range* - *Abbreviations: DME, diabetic macular edema; FAZ, foveal avascular zone; PRI, peripheral retinal ischemia; UWFA, ultra-widefield fluorescein angiography.* | | | | | | | | | | |
